# Nurses’ practice of metabolic monitoring for patients on antipsychotics in Lesotho

**DOI:** 10.4102/sajpsychiatry.v30i0.2219

**Published:** 2024-04-22

**Authors:** Relebohile P. Matete, Libuseng M. Rathobei

**Affiliations:** 1 Department of Nursing, Faculty of Health Sciences, National University of Lesotho, Maseru, Lesotho

**Keywords:** nurse, metabolic monitoring, antipsychotic medication, patients, health care institution

## Abstract

**Background:**

Severe mental illness is associated with higher physical health morbidities and reduced life expectancy, with an estimated 14.3% of global deaths attributed to mental disorders. Antipsychotic medications (APs) used in treatment contribute to physical health issues, including metabolic and cardiovascular effects.

**Aim:**

The aim of this study was to assess nurses’ practices regarding metabolic monitoring for patients prescribed antipsychotic medications at Mohlomi Hospital in Lesotho.

**Setting:**

The study was conducted at Mohlomi Hospital, the primary psychiatric facility in Lesotho.

**Methods:**

Using a cross-sectional design, 44 nurses from Mohlomi Hospital participated in the study. A structured questionnaire assessed nurses’ metabolic monitoring practices.

**Results:**

Most of the respondents were female (*n* = 30, 75%), and minority were male (*n* = 10, 25%). The academic qualifications of respondents were distributed as follows: 40% (16) held a nursing assistant certificate and 22.5% (9) held an advanced nursing diploma, among others. The average age of all respondents was 39.05 (s.d. 8.9), with an average of 8 years of experience in psychiatry (s.d. 7.6). The overall rate of nurses’ practices of metabolic monitoring for patients taking antipsychotic medications showed variability, with a mean score of 2.83 (s.d. 0.524). However, only 20% performed ECG tests, 22.5% measured blood pressure, 27.5% tested for glucose abnormalities and 17.5% conducted lipid profile testing.

**Conclusion:**

Results revealed a significant gap in the practice of metabolic monitoring among nurses with various aspects of metabolic monitoring, not being adequately monitored.

**Contribution:**

The study’s findings shall inform policy and guidelines for monitoring patients on antipsychotic medications while guiding future research.

## Introduction

There is increasing evidence that people with severe mental illness experience more physical health morbidities and have a shorter life expectancy than the general population.^1,2^ Furthermore, Walker et al.^3^ assert that 14.3% of deaths worldwide, or approximately 8 million deaths each year, are attributable to mental disorders. These occurrences may be linked to the evidence that side effects of antipsychotic medications (APs) can increase the risk of developing physical health conditions, and such effects include extrapyramidal effects, dry mouth, metabolic disorders and cardiovascular effects.^4,5^

Consequently, it is of paramount importance that nurses engage in metabolic monitoring for patients taking antipsychotic medications to secure the safe and efficacious use of antipsychotic medications.^6^ Additionally, Coughlin and colleagues^6^ described metabolic monitoring as a comprehensive health assessment for antipsychotic users to detect and prevent cardiovascular and endocrine side effects such as weight gain, hyperglycaemia and arrhythmias. This aligns with the United Kingdom’s National Collaborating Centre for Mental Health, 2014 guideline, where metabolic monitoring for antipsychotic users includes pre-treatment ECG and assessments for weight, waist circumference, blood pressure, glucose, HbA1c, lipids, prolactin and movement disorders.^7^

Additionally, the practice of metabolic monitoring remains scant among nurses across the UK, where a very low proportion of patients diagnosed with psychosis have undergone annual screening and monitoring for metabolic syndrome risk factors such as blood pressure (2%), weight (0%), waist circumference (0%), glucose (7%) and lipid profile (4%).^8^ Similarly, the study in South India found that the monitoring of antipsychotics for metabolic parameters was not in accordance with the American Diabetic Association/American Psychiatric Association (ADA/APA) guidelines and the rate of monitoring remained low, where annual monitoring rates for blood pressure and fasting blood sugar decreased to 72.7% and 46%, respectively, while weight assessments, initially at 60%, decreased to 9.8% for four times the annual assessment.^9^

In sub-Saharan African countries, there is a concerning lack of routine metabolic monitoring following antipsychotic pharmacotherapy, with fewer than 1% of individuals receiving adequate screening.^10^ Consequently, a study conducted in Botswana revealed a moderately high prevalence of metabolic dysfunctions, at 22.6% (95% confidence interval [CI] 15.9, 30.6), among inpatients with severe mental illness, underscoring the importance of assessing cardiovascular risk and targeting modifiable risk factors as suggested to address these figures.^11^ Similarly, a study conducted in South Africa revealed alarmingly low rates of metabolic monitoring among patients with severe mental illnesses, where only 0.6% of patients were screened for abdominal obesity, 3.9% for hyperglycaemia and 1.8% for hypertriglyceridaemia, and merely 0.6% were screened for all these components collectively.^12^ Metabolic monitoring including glucose monitoring has to be part of routine care in hospitals.^13^ Additionally, the minimum acceptable standard is that all patients with mental illness should have their metabolic parameters assessed at the frequency of at least once every 6 months.^14^ In light of this, there is currently no evidenced guideline in the context of Lesotho for metabolic monitoring for psychiatric patients. However, in Lesotho, there are no guidelines regarding metabolic monitoring of patients diagnosed with psychosis therefore, according to the anecdotal evidence, nurses do not perform metabolic monitoring on patients diagnosed with psychosis and patients diagnosed with psychosis continue to experience the detrimental side effects of medications. Therefore, this study holds significance as it aims to describe nurses’ practices regarding metabolic monitoring for patients using antipsychotic medications at a selected health care institution.

## Research methods and design

### Study design

A quantitative, non-experimental, descriptive, cross-sectional design was utilised in this study to obtain information that describes ‘what exists’ with regard to nurses’ practices regarding metabolic monitoring for patients using antipsychotic medications.

### Setting

The study was conducted at Mohlomi Hospital in the Maseru district, located near Makoanyane Barracks in Lesotho. Mohlomi Hospital is the sole psychiatric facility in Lesotho, with a 115-bed capacity. The hospital is divided into various sections, including female and male wards, an outpatient department, a forensic department, a geriatric ward and a child and adolescent ward.

### Population and sampling strategy

The study included 44 nurses from Mohlomi Hospital in 2023. Nurses who met the inclusion criteria were selected as respondents (*N* = 44). This group consisted of 18 nursing assistants, 15 registered nurses with general nursing and midwifery qualifications, and 11 registered nurses specialising in psychiatric nursing with experience of more than 2 years managing patients on antipsychotic medications. Individuals who were not nurses or lacked more than 2 years of experience in managing psychiatric patients on antipsychotic medications were excluded from the study. As Mohlomi Hospital is the sole psychiatric facility in the country, a non-probability convenience sampling method was employed to select participants. This method involves selecting readily available elements during the study period.^15^

### Data collection

The data collection phase spanned 2 weeks during which the researcher distributed questionnaires to available and accessible nurses at Mohlomi Psychiatric Hospital. Out of the 44 nurses employed at the hospital from 03 March 2023 to 17 March 2023, 40 successfully completed the questionnaire and were included in the study. The questionnaire, developed by the researcher with input from a research supervisor and statistician and guided by relevant literature, utilised a Likert scale with five responses: strongly agree, agree, neutral, disagree and strongly disagree. Face validity was established by consulting experts in psychiatry, supervisors and the statistician for feedback on questionnaire validity. To ensure test-retest reliability, the questionnaire was pretested on 10 respondents, and no changes were made based on these data, and the results were not included in the main study findings. The instrument’s (questionnaire) reliability was assessed using Cronbach’s alpha, yielding a score of 0.82 (82%), indicating the reliability of the study’s findings.

Upon obtaining authorisation from the Mohlomi Hospital Nursing Manager and the Ministry of Health in Lesotho (reference number: ID40-2023), the researcher distributed questionnaires to all hospital wards. Data collection spanned 2 weeks, covering day and night shifts, ensuring the participation of nurses across different shifts. Respondents anonymously submitted their questionnaire responses into a designated box. The researcher collected completed forms after 2 weeks, including those from night shift nurses who may have been absent initially. Data security was a priority, with the data file stored securely for 5 years on a server accessible only to the researcher and research supervisor.

### Data analysis

Descriptive statistics play a crucial role in addressing research questions, especially in studies with a primarily descriptive focus. They serve as the foundation for comprehending quantitative research findings.^16^ After collecting completed questionnaires, we tallied and coded them for efficient data capture and auditing. To accommodate negative questions, adjustments were made. Data entry and analysis were performed using Statistical Package for Social Sciences (SPSS), version 28. Descriptive statistics, including percentages, frequencies, means and standard deviations (s.d.), were used to summarise nurses’ practices regarding metabolic monitoring for patients using antipsychotic medications, enhancing interpretation with tables and graphs. Composite scores were computed for questions with varying ranges.

### Ethical considerations

Adherence to at least three ethical principles in health science research has been brought forth: beneficence, respect for human dignity and justice.^17^ The study received approval from the Ministry of Health in Lesotho (Reference No.: ID40-2023) and the Institutional Review Board (IRB) of the National University of Lesotho (Protocol Reference No.: NUL/NUR/2023/16). To ensure participants’ rights, the researcher emphasised voluntary participation, with the option to withdraw consent at any time without consequences. It was clarified that participation would not harm respondents. Informed consent was obtained from willing participants after providing necessary study information. Respondents were assured of anonymity and confidentiality through the use of codes instead of names. All participants were treated equally. Data were securely stored and accessible only to the researcher and supervisor. The study’s findings were described as beneficial to nurses, including non-participants, in enhancing their skills and approaches to patients on antipsychotic medications. Additionally, the study’s results may inform and influence institutional policies.

## Results

### Demographic data

The majority of respondents were female (*n* = 30, 75%), with a minority being male (*n* = 10, 25%). In terms of academic qualifications, respondents were distributed as follows: 40% (16) held a nursing assistant certificate, 32.5% (13) possessed a general nursing diploma, 5% (2) had a nursing degree and 22.5% (9) held an advanced nursing diploma. The average age of all respondents was 39.05 (s.d. 8.9), with an average of 8 years of experience in psychiatry (s.d. 7.6).

### Nurses’ practice of metabolic monitoring

The majority of the respondents (57.5%: agreed or strongly agreed) showed the highest level of agreement towards enquiring about any personal or family history of obesity, diabetes cardiovascular disease and personal history of smoking of patients. Additionally, the statement with the highest level of disagreement was, ‘I routinely draw blood from patients for testing levels of serum prolactin’, where a greater proportion of the respondents (65%) disagreed or strongly disagreed with routinely drawing blood from patients for testing levels of serum prolactin. However, the highest level of uncertainty was seen in the practice of sharing information about the patient’s risk for metabolic syndrome with the multi-disciplinary team, where 55.0% of the respondents were not sure.

The findings generally show a trend of uncertainty or disagreement by the respondents regarding the routine execution of metabolic monitoring practices. The above trend is seen in the findings presented in Table 1.

**TABLE 1:** Nurses’ practice of metabolic monitoring.

Practice section	Strongly agree		Agree		Neutral		Disagree		Strongly disagree		Mean	s.d.
	%	*n*	%	*n*	%	*n*	%	*n*	%	*n*		
I continuously educate patients about their risk for metabolic syndrome	7.5	3	32.5	13	42.5	17	17.5	7	0.0	0	3.30	0.853
I share information about the patient’s risk for metabolic syndrome with the multi-disciplinary team	7.5	3	22.5	9	55.0	22	22.5	9	7.5	3	3.08	0.944
I routinely enquire about any personal or family history of obesity, diabetes cardiovascular disease and personal history of smoking of patients	12.5	5	45.0	18	32.5	4	10.0	4	0.0	0	3.60	0.841
I weigh patients routinely throughout their contact with our services	7.5	3	15.0	6	15.0	6	55.0	22	5.0	2	2.64	1.063
I routinely test patients for glucose abnormalities (i.e. glucose in urine)	12.5	5	10.0	4	17.5	7	50.0	20	10.0	4	2.64	1.063
I routinely measure blood pressure of patients	10.0	4	12.5	5	22.5	9	52.5	21	2.5	1	2.75	1.056
I routinely measure waist circumference of patients	5.0	2	10.0	4	17.5	7	57.5	23	10.0	4	2.43	0.984
I periodically obtain ECG tests for patients	10.0	4	2.0	1	18.0	8	41.0	21	29.0	16	2.28	1.198
I routinely draw blood from patients for testing levels of serum prolactin	10.0	4	7.5	3	17.5	7	40.0	16	25.0	10	2.28	1.198
I routinely conduct suicide risk assessments on patients	10.0	4	25.0	10	30.0	12	25.0	10	10.0	4	3.00	1.155
I conduct assessments to rule out dry mouth in patients	15.0	6	17.5	7	17.5	7	40.0	16	10.0	4	2.88	1.265

## Discussion

### Demographic data

In the current study, the majority of respondents were female, with males forming a smaller group. This contrasts with the findings in the study of Al-Salhee et al.,^18^ where male nurses constituted 58.0% of participants, outnumbering females at 42.0%. Regarding qualifications, most participants in our study held nursing certificates, followed by nursing diplomas, and only a few had advanced nursing diplomas. Conversely, half of the respondents from the work of Al-Salhee et al.^18^ had nursing diplomas. Furthermore, the average age of participants in our study was 39 years, while the majority of participants from the work of Al-Salhee et al.^18^ fell within the 30–39 years age range. This highlights differences in participant demographics between the two studies.

### Nurses’ practice of metabolic monitoring on patients taking antipsychotic medications

A greater proportion of the respondents in the current study showed to engage in educating patients about their risk for metabolic syndrome. This is in line with the study conducted by Zheng and colleagues^19^ which reported that nurse-led lifestyle interventions on metabolic syndrome revealed improvements in self-efficacy to health promotion behaviours. Furthermore, Zhang et al.^20^ reported that nurse-led transition care programme among psychiatric patients with coronary artery disease showed significant effects on self-efficacy.

Additionally, most of the respondents in the current study were uncertain towards sharing information about the patient’s risk of metabolic syndrome with a multi-disciplinary team. This is consistent with the study, which showed that the management of patients diagnosed with metabolic syndrome should include a paradigm shift from a reactive strategy to a proactive approach and the integration of territorial, hospital and social assistance services according to the Chronic Care Model (CCM).^21^ This uncertainty about sharing the information may transcend to inadequate detection of metabolic syndrome in the patients, as well as poor management.

Most of the respondents from this study routinely inquire about any personal or family history of obesity, diabetes, cardiovascular disease and personal history of smoking of patients. This is congruent with the study conducted between 2007 and 2015, where a total of 44 563 respondents received routine health check-ups at the health promotion centre of Tri-Service General Hospital in Taiwan.^22^ Moreover, the study further showed that trained nursing staff collected medical history, family history as well as current medications from each of the participants.^22^

For the practice of weighing patients routinely throughout their contact with our services, most of the respondents in this study disagreed with the routine practice of this assessment. This aligns with the longitudinal study, which reported that a very low proportion of patients diagnosed with psychosis were found to have undergone annual screening and monitoring for metabolic syndrome risk factors such as blood pressure (2%), weight circumference (0%), any glucose (7%) and lipid profile (4%).^22^ This outcome can be linked with inadequate resources in mental health settings and general poor mental health prioritisation in Lesotho by stakeholders.

More than half of the respondents in the current study disagreed with routinely testing patients for glucose abnormalities. This is in contrast with the study on quality improvement initiative undertaken in an inpatient psychiatric ward, and the results revealed rates of glucose monitoring to have increased from 74% to 82.5% (*p* = 0.8256). In addition, the study stated that nurses should continuously monitor glucose and lipid levels every 3 months, depending on whether previous levels were abnormal.^23^ This suggests a need to bring forth workshops to capacitate and sensitise staff about the importance of glucose monitoring for patients taking antipsychotic medications.

Additionally, a greater proportion of the respondents disagreed with routinely measuring the blood pressure of patients. This is in contrary with the study conducted by Taylor^23^ in which the improvement in the appropriateness of monitoring was found for 4 of 5 metabolic parameters after protocol implementation, including blood pressure (17.5% – 43.8%, *p* < 0.001), weight (17.5% – 43.8%, *p* < 0.001), haemoglobin A1C (27.5% – 42.5%, *p* = 0.044) and lipid levels (17.5% – 31.3%, *p* = 0.04).

Moreover, the majority of respondents disagreed with routinely measuring the waist circumference of patients. This is in line with the study conducted by Michael et al.,^24^ which stated a very low proportion of patients diagnosed with psychosis had undergone annual screening and monitoring of waist circumference.

Additionally, the current study found that most respondents do not periodically obtain ECG tests for patients. This study is in contrary with the evidence from in inpatient ward setting, which reported that the clinic led to a statistically significant increase in the number of examinations, blood tests and ECGs successfully obtained.^25^ This difference may be because of the poor resources and incompetent human resources, regarding ECG usage in this setting.

Furthermore, the current study revealed that the majority of respondents do not engage in routine drawing blood from patients for testing levels of serum prolactin. This differs from an enquiry where the results revealed that trained nurses obtained blood samples in the morning after overnight fasting and blood levels of prolactin were measured using electrochemiluminescence immunoassays.^26^

In addition, a greater proportion of the respondents in the current study disagreed and were uncertain about their engagement in suicide risk monitoring among patients. This is in line with the study conducted by Jansson et al.,^27^ cited in Derblom et al.,^28^ which described suicide risk assessment as a demanding task for nurses who feel responsible for another person’s life. Moreover, Jansson et al.,^27^ cited in Derblom et al.,^28^ stated that newer nurses found suicide risk assessment frightening and stated that it was difficult to talk with patients about suicidal thoughts.

Furthermore, the majority of respondents in the current study disagreed with conducting assessments to rule out dry mouth in patients. This is in contrast with the study that indicated that nurses need to assess oral health in somatic screening in order to improve oral health-related quality of life in patients diagnosed with a psychotic disorder.^29^ In addition, the study conducted by Wright and associates^30^ stated that lack of routine oral health examination and treatment in mental health care facilities has been reported as a problem both by persons living with mental illness and psychiatric nurses.

### Limitations

The sample size for this study was limited (*n* = 40) because of the fact that Mohlomi Hospital is the sole psychiatric hospital in Lesotho. As a result, caution must be exercised when attempting to generalise the findings.

### Strengths of the study

The study assessed the practice among nurses who have direct encounters with patients diagnosed with psychosis and also manage their medications. Furthermore, the study was conducted at the one and only tertiary psychiatric hospital in Lesotho, which also serves as the main country-wide referral. This allows for adequate assessment of the study phenomena for the case of Lesotho.

### Implications or recommendations

#### Future research

More qualitative studies need to be conducted to understand the reasons behind the deficiency in the practice of metabolic monitoring for patients on antipsychotic medications.

#### Practice

Implementation of clear clinical guidelines for metabolic monitoring in psychiatric nursing, fostering interdisciplinary collaboration, ensuring patient education and establishing quality assurance measures.

## Conclusion

Based on the findings of this study, it is evident that there is a significant gap in the practice of metabolic monitoring among nurses. The study reveals that various aspects of metabolic monitoring, such as ECG tests, blood pressure, weight, waist circumference, glucose levels and lipid profiles, are not being adequately monitored in patients on antipsychotic medications.
